# Safety of Dabigatran as an Anticoagulant: A Systematic Review and Meta-Analysis

**DOI:** 10.3389/fphar.2021.626063

**Published:** 2021-02-02

**Authors:** Ya Zhou, Zhihao Yao, Linjie Zhu, Yong Tang, Jie Chen, Jianming Wu

**Affiliations:** ^1^ School of Pharmacy, Southwest Medical University, Luzhou, China; ^2^ Affiliated Hospital of Stomatology, Southwest Medical University, Luzhou, China; ^3^ Institute of Cardiovascular Research, The Key Laboratory of Medical Electrophysiology, Ministry of Education of China, Collaborative Innovation Center for Prevention and Treatment of Cardiovascular Disease of Sichuan Province, Medical Key Laboratory for Drug Discovery and Druggability Evaluation of Sichuan Province, Luzhou Key Laboratory of Activity Screening and Druggability Evaluation for Chinese Materia Medica, Luzhou, China; ^4^ Clinical School of Medicine, Southwest Medical University, Luzhou, China

**Keywords:** dabigatran, direct thrombin inhibitor, safety, bleeding, meta-analysis

## Abstract

**Background:** Dabigatran is a univalent low-molecular-weight direct thrombin inhibitor which was developed as an alternative to vitamin K antagonists (VKAs). However, the safety of dabigatran remains controversial so far. In this study, we aimed to compare the risk of bleeding, fatal adverse events, and the all-cause mortality of dabigatran with those of the control group by a systematic review and meta-analysis of randomized controlled trials.

**Methods:** We systematically searched PubMed, Web of Science, Cochrane Library, Medline, Embase, Wanfang database, Clinical trial, China National Knowledge Infrastructure Chinese Scientific Journal database (VIP), and Chinese Biological Medicine database (CBM), for clinical trials on conventional treatments compared with dabigatran, published between January 2014 and July 2020. The reported outcomes, including the endpoints of primary safety, were systematically investigated.

**Results:** Seven RCTs (*n* = 10,743) were included in the present systematic review. Compared to the control groups, dabigatran was not associated with an increased risk of major bleeding (relative risk [RR] 0.86, 95% confidence interval [CI]: 0.61 to 1.21, *p* = 0.06), intracranial hemorrhage (RR 0.89, 95% CI: 0.58 to 1.36, *p* = 0.41), fatal adverse reactions (RR 0.87, 95% CI: 0.65 to 1.17, *p* = 0.66), all-cause mortality (RR 0.88, 95% CI: 0.70 to 1.11, *p* = 0.45, I^2^ = 0%), and significantly reduced risk of clinically relevant non-major bleeding (RR 0.96, 95% CI: 0.65 to 1.42, *p* = 0.0007). However, dabigatran is associated with an increased risk of gastrointestinal (GI) bleeding (RR 1.78, 95% CI: 1.02 to 3.13, *p* = 0.05).

**Conclusion:** Dabigatran has a favorable safety profile in terms of major bleeding, intracranial hemorrhage, and life-threatening events, among other safety outcomes. The present study suggested that dabigatran may be a suitable alternative to VKAs as an oral anticoagulant. However, more data are necessary to clarify the incidence of other adverse events and serious adverse reactions.

## 1 Introduction

According to the World Health Organization, cardiovascular diseases (CVDs) are the leading cause of death in the population. Each year, 17.9 million people die from CVDs, accounting for 31% of the world's total mortality rate (Abdullaev et al., 2019). That number is expected to rise to 23.6 million by 2030 (Laslett et al., 2012). Thrombosis is the leading cause of mortality among the top four causes of death worldwide (Wendelboe, 2016; Mackman, 2020). It can be categorized as arterial thrombosis (AT) or venous thrombosis (VT). Patients with AT may have an increased risk of VT (Prandoni et al., 2006; Prandoni, 2009; Mackman, 2020). Venous thrombosis is caused by endothelial dysfunction due to vessel injury and inflammation or overexpression of thrombogenic factors creates a procoagulant surface (Wolberg et al., 2015). Venous thrombi mostly occur in the deep veins of the legs and arms. The thrombi can break off, travel to the lungs, and lodge in the pulmonary arteries; this process is referred to as pulmonary embolism (PE). Patients with atrial fibrillation (AF) are at a greater risk of stroke owing to the occurrence of larger blood clots than those without AF (Mackman, 2020). Therefore, thrombosis is a fatal risk factor for the health of the patients. Thrombosis leads to numerous diseases, and can be a heavy burden if left untreated. Therefore, the cornerstones of therapeutic strategies for thrombosis are rapid diagnosis and appropriate treatment (Jafarzadeh-Esfehani, 2020).

There are three categories of antithrombotic agents, namely, antiplatelet agents, anticoagulants, and fibrinolytic agents. Anticoagulants are the first-line therapy for the prevention and treatment of VT. There are four classes of anticoagulants, namely, heparins (including low-molecular-weight heparin (LMWH), vitamin K antagonists (VKAs; including warfarin), direct thrombin inhibitors (DTIs; including bivalirudin and dabigatran), and direct FXa inhibitors (FXa; including edoxaban and rivaroxaban) (Mackman, 2020). VKAs, including warfarin, are currently the most commonly used treatment for patients with AF, for the prevention of stroke and venous embolisms, including PE. However, warfarin is associated with a high risk of serious hemorrhagic complications, especially in the elderly. Therefore, treatment with warfarin requires frequent monitoring, and the efficacy of warfarin depends on the nutritional status of the patient (Duan and Jingli, 2020; Song and ZiKai, 2020).

Dabigatran is a reversible DTI with rapid and predictable anticoagulant effects, which do not need of coagulation monitoring and dose adjustments, and does not cause dietary restrictions for patients. Dabigatran etexilate (DE), hereafter referred to as dabigatran, is a small molecule that is orally absorbed after oral administration and converted into dabigatran acting directly by inhibiting thrombin, responsible for the conversion of fibrinogen into fibrin during coagulation cascade and preventing the development of thrombus (clot). (Yasaka et al., 2013; Pepe Ribeiro de Souza C et al., 2015).

Dabigatran treatment has been shown to be cost-effective compared with warfarin treatment, with better clinical results and an additional cost justified by the benefit in terms of overall survival and quality of life provided to the patient (Pepe Ribeiro de Souza C et al., 2015). DE has a wide range of clinical applications. The efficacy of DE has been demonstrated in several clinical studies for the prevention of venous thromboembolism (VTE) in patients undergoing total hip or knee replacement, for the prevention of stroke in patients with nonvalvular AF, and in treating acute VTE (Di Biase et al., 2014; Gombár et al., 2014; Diener et al., 2015; Ferner et al., 2016; Kim et al., 2016).

An existing meta-analysis was published in 2013 to compare the risk of bleeding and all-cause mortality of dabigatran with that of VKAs in a systematic review and meta-analysis of RCTs. But since 2014, many researchers have questioned the safety of dabigatran. Clinical trials have also demonstrated that the use of novel oral anticoagulants (NOACs) is associated with an increased risk of gastrointestinal (GI) bleeding compared with VKAs (Holster et al., 2013). A meta-analysis of randomized controlled trials (RCTs) additionally suggested that DE significantly increases the risk of GI bleeding compared with VKAs (relative risk (RR): 1.41, 95% confidence interval (CI): 1.28–1.55; *p* < 0.001) (Sipahi et al., 2014). However, another study confirmed that the risk of upper GI bleeding is lower with DE than with VKAs (Di Minno et al., 2017). Nevertheless, it is unclear whether dabigatran increases the risk of myocardial infarction and massive bleeding. It is argued that dabigatran may reduce the mortality and morbidity associated with VTE, but may also increase the risk of massive bleeding (Majeed et al., 2016; Di Minno et al., 2017; Polzin et al., 2018; Butt et al., 2020; Izcovich et al., 2020; Ma et al., 2020). The aim of this meta-analysis was to retrieve and assemble data from RCTs on the safety of patients receiving anticoagulant therapy with dabigatran.

## 2 Materials and Methods

### 2.1 Search Strategy and Selection Criteria

The systematic review and meta-analysis were performed following the PRISMA guidelines and the Cochrane Handbook. We searched the literature published between January 2014 and July 2020 from ten databases, including PubMed, Web of Science, Cochrane Library, Medline, embase, Wanfang database, Clinical Trials, China National Knowledge Infrastructure (CNKI), Chinese Scientific Journal database (VIP), Chinese Biological Medicine database (CBM), and Clinical Trials (http://www.clinicaltrials.gov). We used the following search strings in all possible combinations: “dabigatran”, “dabigatran etexilate”, “DE”“anticoagulant”, “safety”, “venous embolism”, “venous thrombosis”, “NOACs”, and “DOACs”, without any language restrictions.

The studies that met the following inclusion criteria were considered in this study: 1) RCTs; 2) studies on research subjects aged 18 years or older who needed anticoagulant therapy; 3) studies where treatment with no other medicines, except dabigatran, in combination with conventional therapies in the experimental group was compared to conventional treatments as the control; 4) studies where one or more outcome measures, including severe/major bleeding, myocardial infarction, clinically relevant non-major bleeding, GI bleeding, and adverse events were included.

Studies with the following exclusion criteria were not considered: 1) studies that did not focus on dabigatran; 2) studies with inappropriate criteria in the experimental or control groups; For example, the trial design was not rigorous as, it includes the following six points: a) in terms of the selection of subjects (patients), the inclusion criteria and exclusion criteria in more than half of the clinical trial protocols were unscientific and not rigorous, and the diagnostic criteria of diseases, including the basic conditions of subjects, were not clearly described; b) for interventions, researchers often fail to scientifically and accurately set the drug selection and dosage, or lack unified standard operating procedures for surgical interventions in multi-center studies; c) attention should be paid to comparability and ethics in the control group. It usually was neglected in collecting and organizing informations; d) the researchers did not distinguish the primary end point from the secondary end point, and the endpoint indicators were set in confusion; e) statistical scheme is missing or incorrect; f) the training of test participants, the formulation and implementation of operation norms and guidelines, the follow-up management, the data management and quality control, the test supervision, and the standard record, report and treatment of adverse events and serious adverse events are not strictly managed; 3) studies where the experimental design was not rigorous, a control group was not designed, and the data were incomplete; 4) non-contrast articles, non-clinical studies, literature reviews, meta-analyses, meeting abstracts, case reports, repetitive studies, and studies on experimental models.

### 2.2 Data Extraction and Quality Assessment

The data were independently extracted by two reviewers (Z. Y. and Y. Z. H.) based on the same inclusion and exclusion criteria. Any disagreements were adjudicated by a third investigator (Z. L. J.). The following characteristics were extracted: 1) name of the first author; 2) year of publication; 3) number of cases; 4) ages of the patients; 5) basic characteristics of the patients; 6) intervening measures; 7) dosage of dabigatran; 8) duration of treatment; and 1) types of study parameters. The quality of the included trials was evaluated according to the Cochrane Handbook (Xue et al., 2019).

### 2.3 Definition of Outcomes

The primary safety outcomes of dabigatran as an anticoagulant were the incidence of major bleeding and clinically relevant non-major bleeding. The additional safety outcomes were GI bleeding, non-major bleeding, systemic embolism, and other adverse events. Major bleeding was defined on the basis of the criteria provided by the International Society on Thrombosis and Hemostasis (ISTH) (Schulman et al., 2005; Schulman et al., 2010). Clinically relevant non-major bleeding was defined by the need for hospitalization, medical or surgical interventions, a change, interruption, or discontinuation of the trial drug, and a composite of major or clinically relevant non-major bleeding (Diener et al., 2019). Other adverse events were defined as other mildly unpleasant medical events that occurred in patients receiving the experimental treatment (Calkins et al., 2017).

### 2.4 Statistical Analysis and Assessment of Risk of Bias

All the analyses were performed in the intention-to-treat population, unless otherwise specified. The statistical analyses were performed using Review Manager, version 5.3 (Cochrane Collaboration, Oxford, United Kingdom). As all the data obtained from the studies were comparisons of categorical data, the data were expressed as estimate RR and 95% CI. For the analyses, *p* < 0.05 was considered statistically significant (Di Minno et al., 2017). Heterogeneity among the studies was estimated using the Chi-square test and I^2^ tests. A random-effects model was used to pool the data when I^2^ > 50%, which is indicative of a high statistical heterogeneity, while a fixed-effects model was chosen when I^2^ ≤ 50% (Liang et al., 2018).

Publication bias was numerically examined by Begg’s and Egger’s tests, and the results are graphically represented by funnel plots of the standard difference in the mean vs. the standard error (Di Minno et al., 2017). The asymmetry of the funnel plot was visually assessed to addressing any possible small-study effects, in combination with Egger’s test to addressing the publication bias, over and above any subjective evaluation. *p* < 0.1 was considered to be statistically significant (Sterne et al., 2001). If publication bias existed, the pooled estimates of the potential unpublished studies in the meta-analysis were adjusted by the trim-and-fill method, which were compared with the original pooled Odds Ratio (OR) (Liang et al., 2018; Lin et al., 2018; Xue P, et al., 2019).

## 3 Results

### 3.1 Results of Literature Search

**FIGURE 1:**
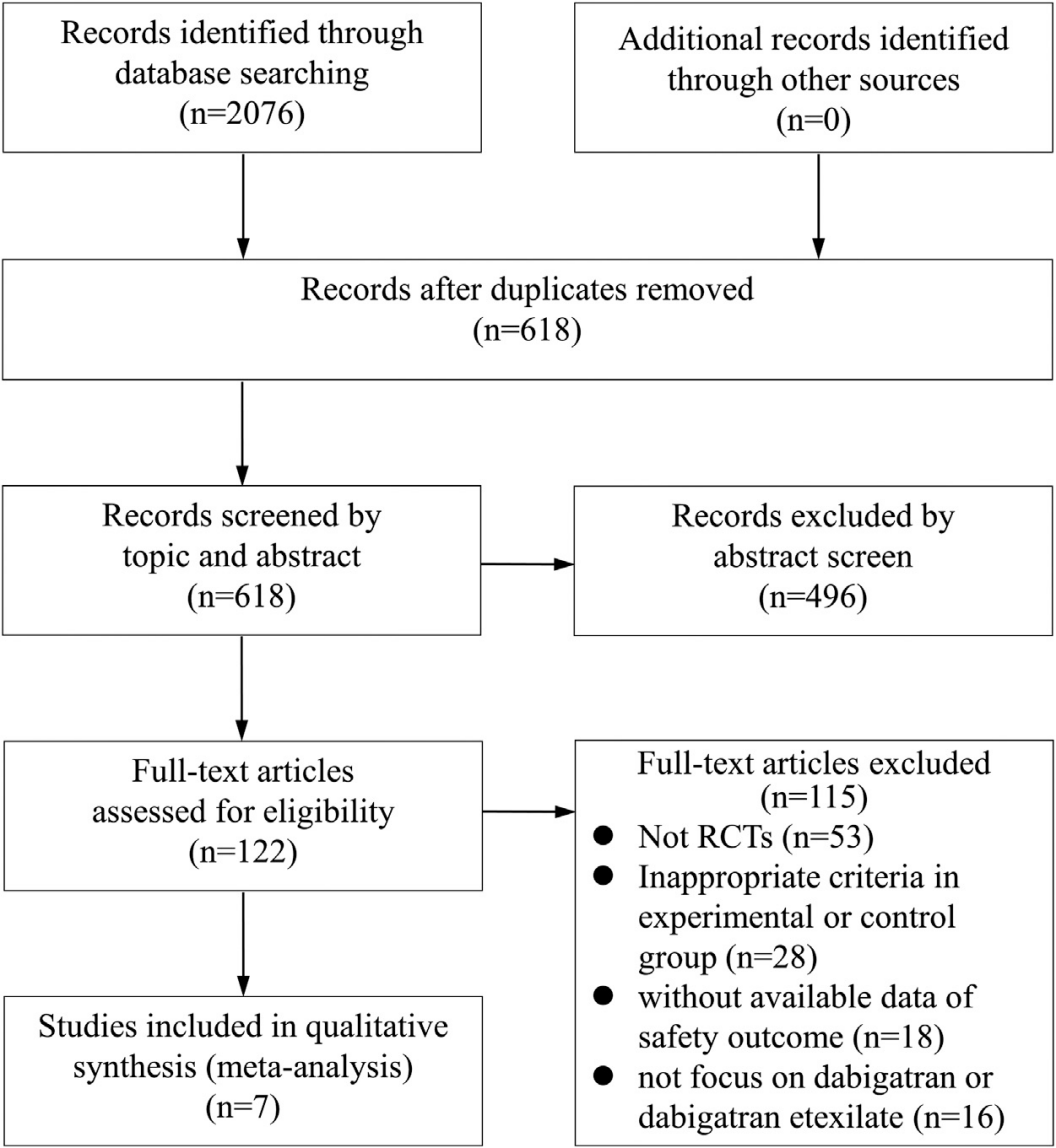
Process of selection of studies for meta-analysis.

A total of 2076 articles were initially selected by literature search. After reviewing the titles and abstracts, a total of 122 studies were found to be potentially relevant. After a careful review of the full texts, seven trials involving 10,743 participants were finally included for the analysis, according to the aforementioned inclusion and exclusion criteria (Figure 1). A total of 5,337, 2,695, 1,606, 168, 60, and 877 patients were randomized to receive dabigatran, aspirin, warfarin, enoxaparin, rivaroxaban, and the placebo, respectively.

**FIGURE 2:**
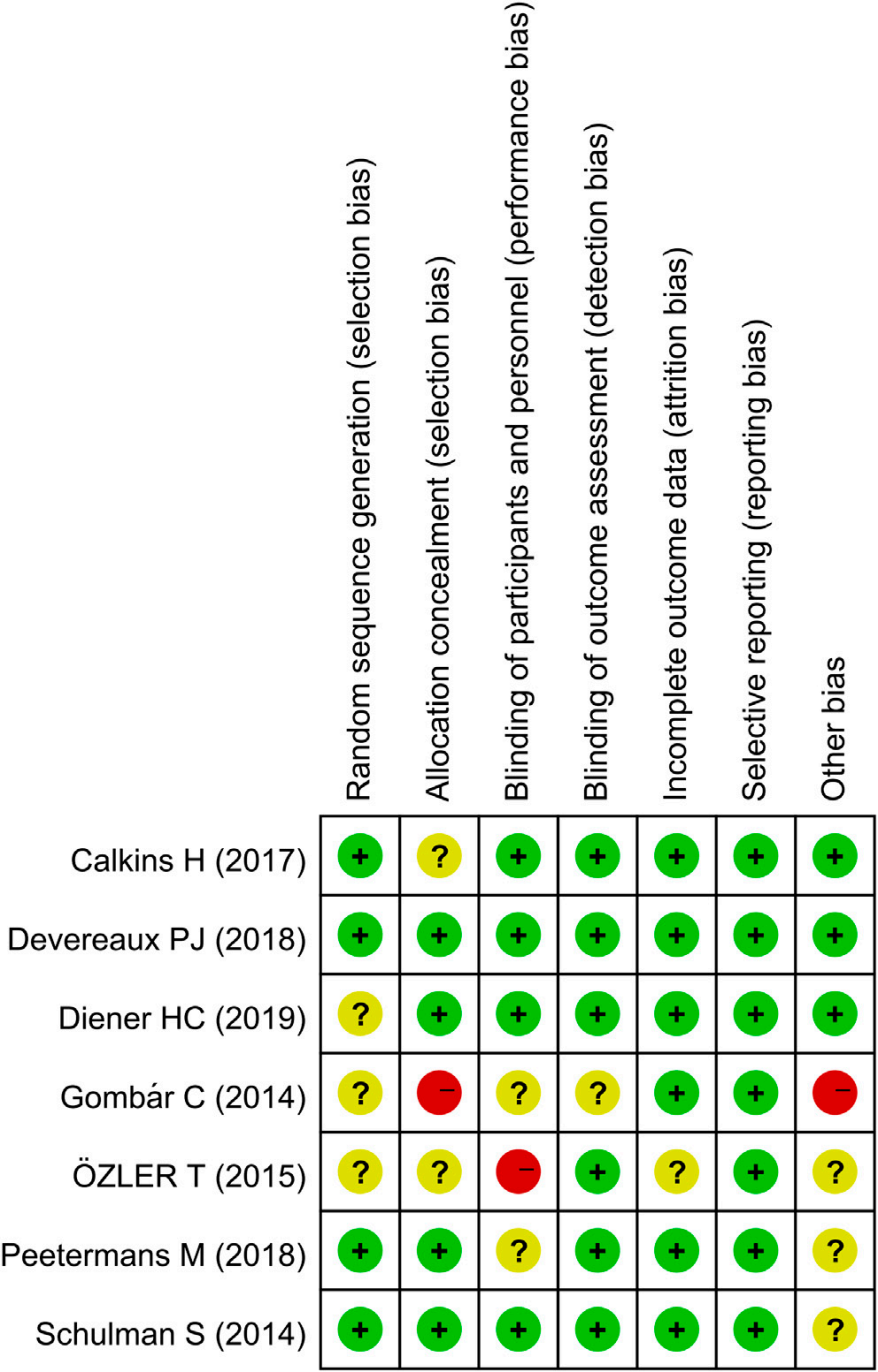
Summary of risk of bias.

The assessment of the risk of bias is depicted in Figure 2. Five studies had a low risk of bias, as defined by the Cochrane tool for evaluating the risk of bias. Two studies were considered to have a high risk of bias owing to unclear randomization, methods for blinding, and allocation concealment.

### 3.2 Patient Characteristics

The trials included in our study were performed in different medical centers across the world. The characteristics of the patients were found to be similar, without any significant differences. All the trials included in this study clearly stated the dosage of dabigatran. The majority of participants in three studies received 150 mg dabigatran twice daily (Schulman et al., 2014; Calkins et al., 2017; Diener et al., 2019). In two studies the patients received 110 mg dabigatran twice daily (Devereaux et al., 2018; Peetermans et al., 2018), while the dose of dabigatran in two other studies was 220 mg, once a day (Gombár et al., 2014; Özler et al., 2015). In all RCTs, the patients were predominantly men, with the exception of one patient (Özler et al., 2015), and the mean age of the patients ranged from 40 to 87 years. The duration of treatment and follow-up ranged from 7 to 990 days. In one study, the patients with coronary heart disease who were assigned to the dabigatran group, received aspirin for the treatment of coronary heart disease, while the patients in the control group (aspirin group) with coronary heart disease received aspirin plus placebo (Diener et al., 2019). In another study, the patients received 2 × 0.3 ml^−1^ enoxaparin during their stay in the hospital, and switch therapy with 1 × 10 mg rivaroxaban 1 × 0.4 ml^−1^ enoxaparin or 1 × 220 mg dabigatran (Özler et al., 2015). The major characteristics of the patients in the studies included herein are depicted in Table 1.

**TABLE 1:** Study and patient characteristics of the seven trials comparing dabigatran with control group.

Included studies	Patients	Age (year) (mean)	Female (%)	Study population	Length of treatment (days)	Length of follow-up (days)	Number of patients lost to follow-up
Diener et al. (2019)				Embolic stroke of undetermined source			
Dabigatran 150^a^	2,695	64.5 ± 11.4	36.6		990	990	19
Aspirin	2,695	63.9 ± 11.4	37.1				14
Devereaux et al. (2018)				Myocardial injury after non-cardiac surgery			
Dabigatran 110^b^	877	70 ± 11	48		720	720	9
Placebo	877	70 ± 11	49				10
Peetermans et al. (2018)				Staphylococcus aureus bacteraemia			
Dabigatran 110^b^	47	64 ± 15	15		7–10	90	10
Enoxaparin	47	63 ± 18	43				9
Calkins et al. (2017)				Ablation of atrial fibrillation			
Dabigatran 150^c^	317	59.1 ± 10.4	27.4		112	7	8
Warfarin	318	59.3 ± 10.3	23				7
Özler (2015)				Total hip arthroplasty (THA) and total knee arthroplasty (TKA)			
Dabigatran 220^d^	60	49–82 (range)	62		10 (TKA) and 30 (THA)	42	NR
Enoxaparin	60	40–87 (range)	63				
Rivaroxaban	60	45–80 (range)	72				
Schulman et al. (2014)				Acute venous thromboembolism			
Dabigatran 150^c^	1,280	54.7 ± 16.2	39		180	30	125
Warfarin	1,288	55.1 ± 16.3	39.8				116
Gombár et al. (2014)				Total hip replacement			
Dabigatran 220^e^	61	69 ± 7.6	28		7	90	0
Enoxaparin	61	69 ± 9.7	26				0

Note: NR, not report.

^a^ Dabigatran was given at a dose of 150 mg twice daily, 110 mg twice daily in ≧75 years old patients.

^b^ Dabigatran was given at a dose of 110 mg twice daily.

^c^ Dabigatran was given at a dose of 150 mg twice daily.

^d^ Dabigatran was given at a dose of 220 mg daily.

^e^ DE was given at a dose of 220 mg daily, 150 mg in ≧75 years old patients.

### 3.3 Safety Outcomes

The safety outcomes of the trials included were grouped into various subcategories in our study (Table 2).

**TABLE 2:** Study and patient characteristics of the seven trials comparing dabigatran with control group.

Included studies	N	Major bleeding	Clinically relevant non-major bleeding	Intracranial hemorrhage	Gastrointestinal bleeding	Fatal adverse event	All-cause mortality
Diener et al. (2019)							
Dabigatran 150^a^	2,695	77(2.9)	145(5.4)	32(1.2)	27(1.0)	39	14(0.52)
aspirin	2,695	64(2.4)	101(3.7)	32(1.2)	22(0.8)	51	23(0.85)
Devereaux et al. (2018)							
Dabigatran 110^b^	877	29(3.3)	26(3.0)	4(0.46)	48(5.5)	11	100(11.4)
Placebo	877	31(3.6)	35(4.0)	3(0.34)	13(1.5)	10	110(12.5)
Peetermans et al. (2018)							
Dabigatran 110^c^	47	1(2.2)	4(8.5)	NR	2(4.3)	NR	10(21.3)
Enoxaparin	47	1(2.2)	4(8.5)	NR	0	NR	9(19.2)
Calkins et al. (2017)							
Dabigatran 150^a^	338	4(1.2)	NR	0	1(0.3)	NR	0
Warfarin	338	21(6.2)	NR	2(0.6)	2(0.6)	NR	0
Özler (2015)							
Dabigatran 220^days^	60	1(1.7)	NR	NR	NR	NR	NR
Enoxaparin	60	1(1.7)	NR	NR	NR	NR	NR
Rivaroxaban	60	0	NR	NR	NR	NR	NR
Schulman et al. (2014)							
Dabigatran 150^a^	1,280	34(2.7)	64(5)	2(0.2)	48(3.8)	0	NR
Warfarin	1,288	45(3.5)	102(7.9)	6(0.5)	33(2.6)	1(0.08)	NR
Gombár et al. (2014)							
Dabigatran 220^e^	61	22(36.1)	34(55.7)	NR	NR	22(36.1)	NR
Enoxaparin	61	21(34.4)	29(47.5)	NR	NR	21(34.4)	NR

Note: Data are presented as No. of patients (%). N = number of patients who had taken at least one dose of trial drug. UD, unable to determine; NR, not report.

#### 3.3.1 Major Bleeding

**FIGURE 3:**
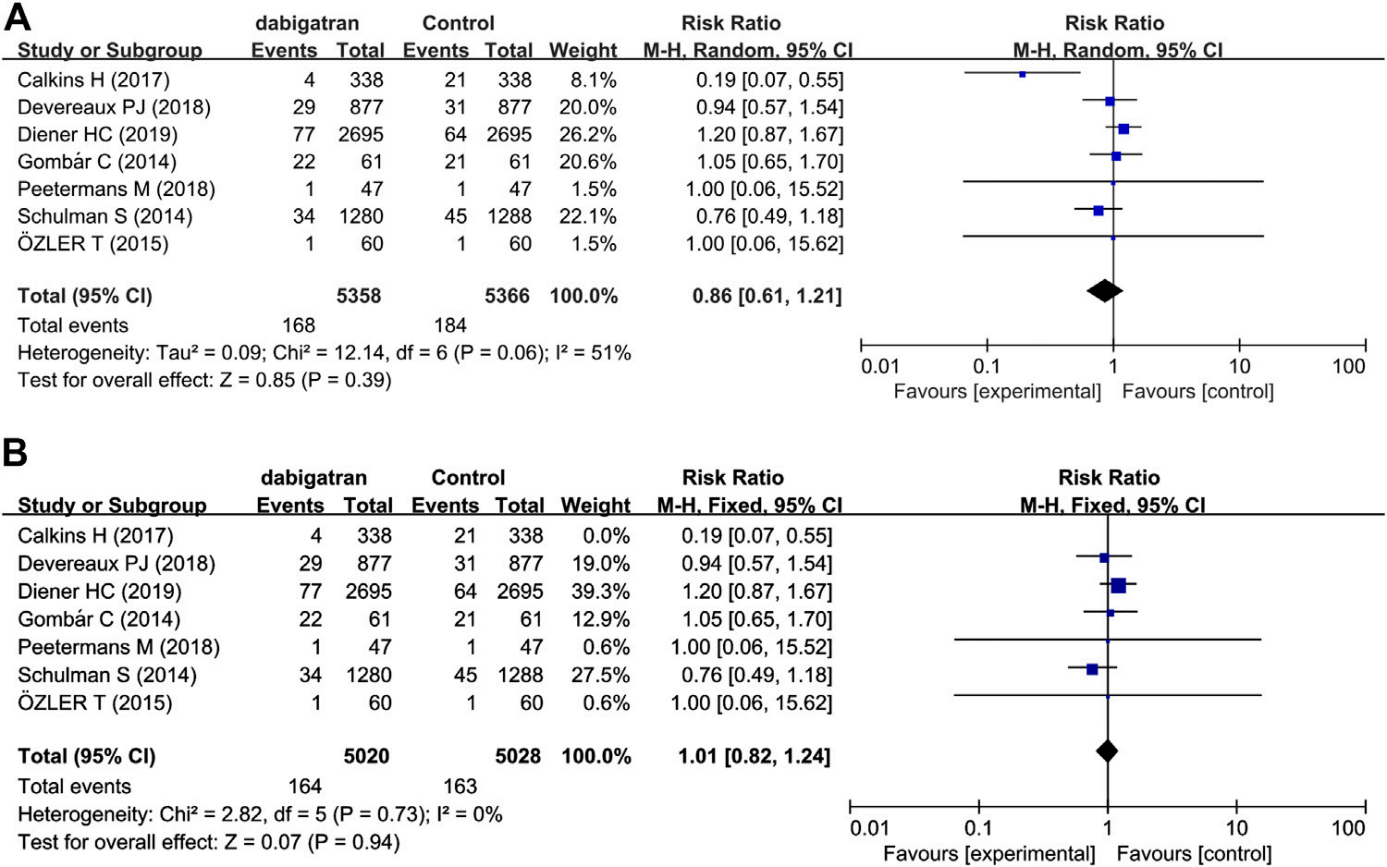
Forest plot comparing the RR of major bleeding between the experimental (dabigatran) and control group **(A)**. Control group, conventional treatments with other medications; experimental group, conventional treatments with dabigatran. Forest plot after heterogeneity was deleted **(B)**.

All the studies reported the incidence of major bleeding based on the criteria provided by the ISTH (Gombár et al., 2014; Schulman et al., 2014; Özler et al., 2015; Calkins et al., 2017; Devereaux et al., 2018; Peetermans et al., 2018; Diener et al., 2019). Major bleeding occurred in 168 (3.1%) of the 5,358 patients receiving dabigatran and in 184 (3.4%) of the 5,366 patients in the control group (RR 0.86, 95% CI: 0.61 to 1.21, *p* = 0.06, I^2^ = 51%; Figure 3). The heterogeneity was high because the results reported in one trial (Calkins et al., 2017) were inconsistent with those of the other studies. Owing to the high heterogeneity, a random-effects model was used to analyze the rate of RR. The risk of major bleeding events did not increase or decrease in the patients who were randomized to dabigatran, compared to that of the patients randomized to the control groups.

#### 3.3.2 Clinically Relevant Non-Major Bleeding

**FIGURE 4:**
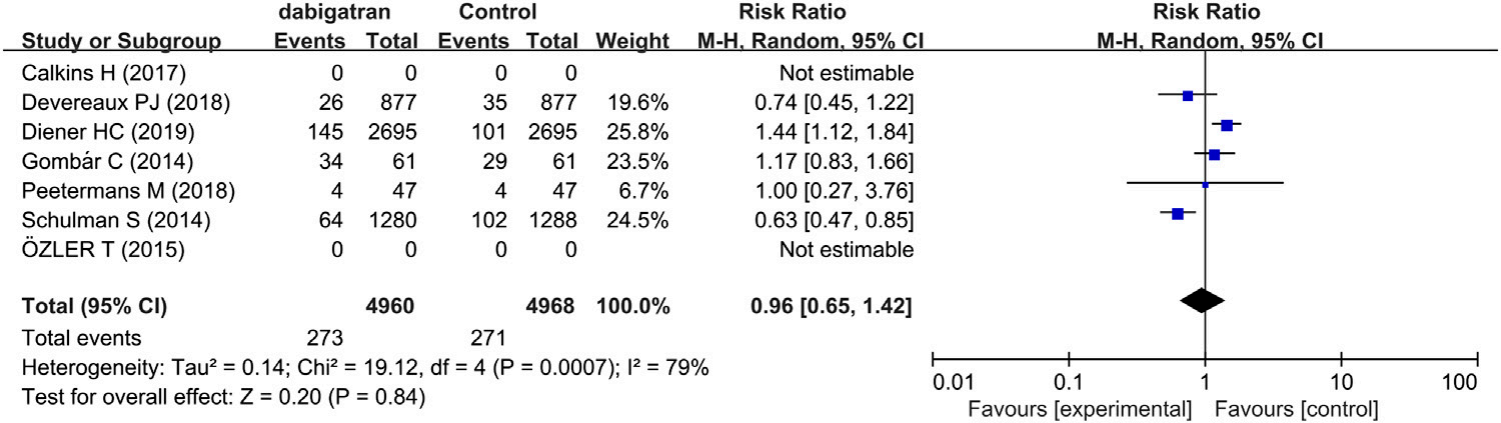
Forest plot comparing the RR of clinically relevant non-major bleeding between the experimental (dabigatran) and control groups.

Five studies reported the incidence of direct and indirect clinically relevant non-major bleeding (Gombár et al., 2014; Schulman et al., 2014; Devereaux et al., 2018; Peetermans et al., 2018; Diener et al., 2019). In each group, the incidence of direct and indirect clinically relevant non-major bleeding in both group was 5.5%. The data showed that there was no difference in the risk of clinically relevant non-major bleeding among the 4,960 patients treated with dabigatran and the 4,968 patients who received other treatments (RR 0.96, 95% CI: 0.65 to 1.42, *p* = 0.0007, I^2^ = 79%; Figure 4). The results of analysis revealed that heterogeneity was derived from two studies, which differed possibly due to differences in the intervention measures of the control group (Schulman et al., 2014; Diener et al., 2019). The risk of clinically relevant non-major bleeding events did not increase or decrease in patients randomized to dabigatran, compared to that of the patients randomized to the control groups.

#### 3.3.3 Intracranial Hemorrhage

**FIGURE 5:**
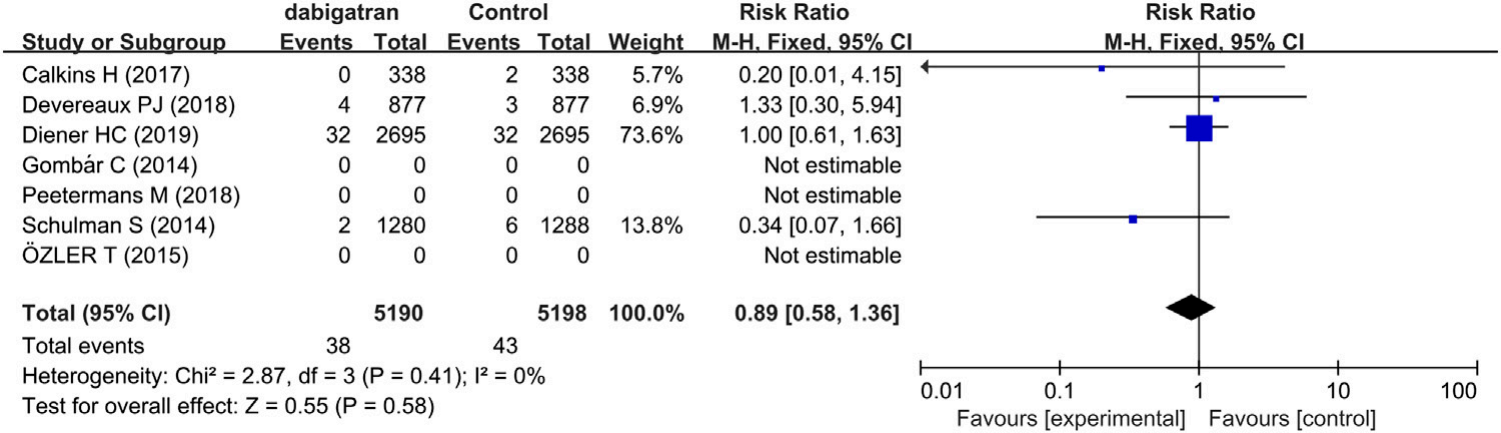
Forest plot comparing the RR of intracranial hemorrhage between the experimental (dabigatran) and control groups.

Analysis of four studies reporting the total number of patients experiencing intracranial hemorrhage revealed that the incidence of intracranial hemorrhage was 0.7% in the patients treated with dabigatran and 0.8% in the control groups, with a corresponding RR of 0.89 (95% CI: 0.58, 1.36, *p* = 0.41, I^2^ = 0%, Figure 5) (Schulman et al., 2014; Calkins et al., 2017; Devereaux et al., 2018; Diener et al., 2019).

#### 3.3.4 Gastrointestinal Bleeding

**FIGURE 6:**
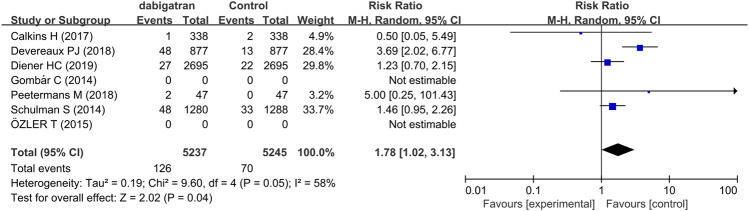
Forest plot comparing the RR of GI bleeding between the experimental (dabigatran) and control groups.

Five trials assessed the risk of GI bleeding. The results demonstrated that the risk of GI bleeding in patients receiving dabigatran was consistently higher and statistically significant compared to that of the patients randomized to the control groups (RR 1.78, 95% CI: 1.02 to 3.13, *p* = 0.05, I^2^ = 58%, Figure 6) (Schulman et al., 2014; Calkins et al., 2017; Devereaux et al., 2018; Peetermans et al., 2018; Diener et al., 2019). The rate of GI bleeding was 2.4% in the dabigatran groups and 1.3% in the control groups.

#### 3.3.5 Fatal Adverse Event

**FIGURE 7:**
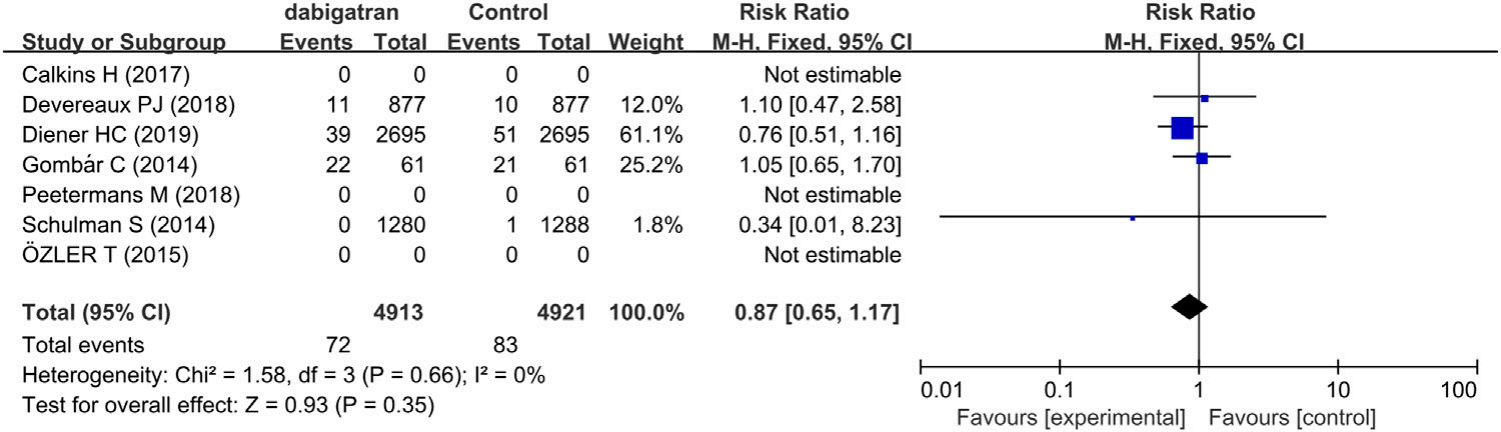
Forest plot comparing the RR of fatal bleeding events between the experimental (dabigatran) and control groups.

Fatal adverse events are a combination of life-threatening bleeding and fatal bleeding events (Table 2). The analysis of four studies revealed that treatment with dabigatran did not increase the risk of fatal adverse reactions (RR 0.87, 95% CI: 0.65 to 1.17, *p* = 0.66, I^2^ = 0%, Figure 7).

#### 3.3.6 All-Cause Mortality

**FIGURE 8:**
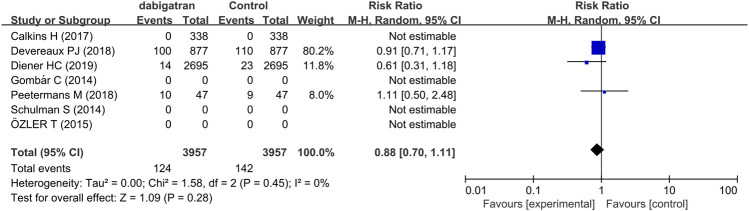
Forest plot comparing the RR of all-cause mortality between the experimental (dabigatran) and control groups.

Three trials reported data regarding the all-cause mortality of dabigatran. The mortality in patients treated with dabigatran tended to be lower (RR 0.88, 95% CI: 0.70 to 1.11, *p* = 0.45, I^2^ = 0%, Figure 8).

## 4 Discussion

The safety of the novel oral anticoagulant dabigatran has been a topic of much discussion. Numerous clinical trials have been conducted to confirming the safety and efficacy of dabigatran. In this study, we aimed to compare the safety profile of the novel oral anticoagulant dabigatran, with that of a control substance. A meta-analysis was conducted with the data published in seven research articles, and the results demonstrated that treatment with dabigatran did not increase the risk of major bleeding and intracranial hemorrhage, and was associated with a decreased risk of clinically relevant non-major bleeding and fatal adverse events. However, these benefits came at the expense of an increased risk of GI bleeding. We also observed that the risk of all-cause mortality tended to be lower than that in the control groups; however, the results were not statistically significant.

In our study, four trials reported the incidence of intracranial hemorrhage, of which only two studies performed a comparative study with warfarin. We found that the risk of intracranial bleeding with dabigatran was lower than that following treatment with VKAs, and the difference was statistically significant. This is a major advantage with dabigatran, as intracranial hemorrhage is a risky and harmful complication (Connolly et al., 2009; Schulman et al., 2009; Bloom et al., 2014). This is beneficial for patients on long-term medication, and improves their quality of life.

We observed that the risk of fatal adverse events and all-cause mortality in patients receiving dabigatran were lower than those of the patients receiving placebo or aspirin. In the trial conducted by Diener and coworkers, one patient (0.02% per year) in the dabigatran group and six patients (1.4% per year) in the aspirin group experienced fatal bleeding and fatal hemorrhagic stroke, which were the primary end points of the study Hazard Ratio (HR) 1.19, 95% CI: 0.85–1.66) (Diener et al., 2019). They also observed that dabigatran is more effective than aspirin for preventing stroke in patients with embolic strokes of undetermined sources, owing to the lower incidence of embolism with dabigatran. The overall reduction in the all-cause mortality could be attributed to the reduced risk of all-cause mortality without any significant increase in the risk of major vascular complications.

Anticoagulant therapy invariably involves a trade-off between fewer thrombotic events and increased bleeding (Devereaux et al., 2018). DE can directly anchor and inhibit free and coagulated thrombin, and block thrombin induced platelet aggregation. Dabigatran oral bioavailability is low and absorption requires an acidic environment, which increases the burden of the GI tract and the risk of GI bleeding (Barton et al., 2012; Schleichert et al., 2016). The results of our study demonstrated an increase in GI bleeding with dabigatran, similar to the results of previous studies (Di Minno et al., 2017). The study (Devereaux et al., 2018) showed the predictable increase in minor and clinically non-significant lower gastrointestinal bleeding with dabigatran. However, another study (Di Minno et al., 2017) reported that dabigatran was consistently associated with a lower risk of upper GI bleeding compared with VKAs. Therefore, we hope that there will be more clinical studies to distinguish between upper GI bleeding and lower GI bleeding to determine the risk of dabigatran, so as to make the research data more accurate.

Our study has some potential limitations. Dabigatran was mainly excreted by kidney, 77% of which was excreted in urine. With the increase of age and the decline of renal function, the clearance time of dabigatran was prolonged. Studies (Barton, et al., 2012; Di Minno et al., 2017) have shown that age, hypertension, diabetes, and renal insufficiency directly affect the bleeding risk of dabigatran, and when combined with drugs, it will increase the incidence of adverse reactions. Therefore, whether dabigatran increases the risk of GI bleeding or other bleeding should be studied at different levels, such as patient's age, underlying diseases, drug interactions, dosage, and renal function (Oldgren et al., 2011; Mega, et al., 2012). In addition, there are some significant differences in clinical and demographic characteristics and dosages of patients enrolled in different studies. With the meta-regression approach we were able to adjust results for some, but not all potential confounders. A further potential source of bias is the difference in the basic treatment, either using placebo, warfarin, or other drugs, which leads to different adverse events in the control group. This may limit the reproducibility of our findings in the general population. However, as our analysis is based on a systematic search of all published studies, without any language restrictions, and through high-quality RCTs and extensive literature review, we are confident that the potential impact of this type of bias on our results will be minimized lowest.

## 5 Conclusion

The results of our large meta-analysis suggest that dabigatran and other anticoagulants have a similar or even lower risk of bleeding. However, due to the limited number of samples and the limited setting conditions, the security of dabigatan needs to be further studied. In anticoagulant therapy, because of the short half-life of dabigatran, the administration may be suspended or delayed according to the patient's bleeding condition. If major bleeding or fatal bleeding occurs, an anticoagulant reversal agent should be used urgently.

## Data Availability Statement

Publicly available datasets were analyzed in this study. This data can be found here: https://www.ncbi.nlm.nih.gov/pubmed.

## Author Contributions

YZ, ZY, and JW conceived and designed the study; YZ, ZY, LZ, YT, JC, and JW reviewed the literature; and YZ, ZY, LZ, and JW wrote the manuscript.

## Funding

This work was supported by grants from National Key Research and Development Program of China (Grant Nos. 2018ZX09721004-006-004); National Natural Science Foundation of China (Grant Nos. 81774013 and 82074129); Science and Technology Planning Project of Sichuan Province, China (Grant Nos. 2019JDPT0010, 2018JY0237, 2019LZXNYDJ11, 2019YJ0484, and 2019YJ0473); Educational Commission of Sichuan Province, China (Grant Nos. 18TD0051 and 18ZA0525); Joint Project of Luzhou Municipal People’s Government and Southwest Medical University, China (Grant Nos. 2020LZXNYDZ03), The Administration of Traditional Chinese Medicine of Sichuan Province, China (Grant Nos. 2018JC013); Foundation of Southwest Medical University, China (Grant Nos. 2017-ZRQN-186).

## Conflict of Interest

The authors declare that the research was conducted in the absence of any commercial or financial relationships that could be construed as a potential conflict of interest.

## References

[B1] Abdullaev S. P. Mirzaev K. B. Sychev D. A. (2019). Comparative clinical and economic evaluation of pharmacogenetic testing application for dabigatran in patients with atrial fibrillation. Ter. Arkh. 91 (8), 22–27 [Russian]. 10.26442/00403660.2019.08.000379 32598750

[B2] Barton C. A. McMillian W. D. Raza S. S. Keller R. E. (2012). Hemopericardium in a patient treated with dabigatran etexilate. Pharmacotherapy 32 (5), e103–e107. 10.1002/j.1875-9114.2012.01036.x 22488474

[B3] Bloom B. J. Filion K. B. Atallah R. Eisenberg M. J. (2014). Meta-analysis of randomized controlled trials on the risk of bleeding with dabigatran. Am. J. Cardiol. 113 (6), 1066–1074. 10.1016/j.amjcard.2013.11.049 24440332

[B4] Butt J. H. Fosbøl E. L. Verhamme P. Gerds T. A. Iversen K. Bundgaard H. (2020). Dabigatran and the risk of staphylococcus aureus bacteremia: a nationwide cohort study. Clin. Infect. Dis. [Epub ahead of print]. 10.1093/cid/ciaa661 32478836

[B5] Calkins H. Willems S. Gerstenfeld E. P. Verma A. Schilling R. Hohnloser S. H. (2017). Uninterrupted dabigatran versus warfarin for ablation in atrial fibrillation. N. Engl. J. Med. 376 (17), 1627–1636. 10.1056/NEJMoa1701005 28317415

[B6] Connolly S. J. Ezekowitz M. D. Yusuf S. Eikelboom J. Oldgren J. Parekh A. (2009). Dabigatran versus warfarin in patients with atrial fibrillation. N. Engl. J. Med. 361 (12), 1139–1151. 10.1056/NEJMoa0905561 19717844

[B7] Devereaux P. J. Duceppe E. Guyatt G. Tandon V. Rodseth R. Biccard B. M. (2018). Dabigatran in patients with myocardial injury after non-cardiac surgery (MANAGE): an international, randomised, placebo-controlled trial. Lancet 391 (10137), 2325–2334. 10.1016/S0140-6736(18)30832-8 29900874

[B8] Di Biase L. Burkhardt J. D. Santangeli P. Mohanty P. Sanchez J. E. Horton R. (2014). Periprocedural stroke and bleeding complications in patients undergoing catheter ablation of atrial fibrillation with different anticoagulation management: results from the role of coumadin in preventing thromboembolism in atrial fibrillation (AF) patients undergoing catheter ablation (COMPARE) randomized trial. Circulation 129 (25), 2638–2644. 10.1161/CIRCULATIONAHA.113.006426 24744272

[B9] Di Minno M. N. Ambrosino P. Di Minno A. Tremoli E. Di Minno G. (2017). The risk of gastrointestinal bleeding in patients receiving dabigatran etexilate: a systematic review and meta-analysis of the literature. Ann. Med. 49 (4), 329–342. 10.1080/07853890.2016.1268710 28084107

[B10] Diener H. C. Easton J. D. Granger C. B. Cronin L. Duffy C. Cotton D. (2015). Design of randomized, double-blind, evaluation in secondary stroke prevention comparing the efficacy and safety of the oral Thrombin inhibitor dabigatran etexilate vs. acetylsalicylic acid in patients with embolic stroke of undetermined source (RE-SPECT ESUS). Int. J. Stroke 10 (8), 1309–1312. 10.1111/ijs.12630 26420134

[B11] Diener H. C. Sacco R. L. Easton J. D. Granger C. B. Bernstein R. A. Uchiyama S. (2019). Dabigatran for prevention of stroke after embolic stroke of undetermined source. N. Engl. J. Med. 380 (20), 1906–1917. 10.1056/NEJMoa1813959 31091372

[B12] Duan J. Yang L. Li H. Yamamura N. Harada A. (2020). Pharmacokinetics and safety of dabigatran etexilate after single and multiple oral doses in healthy Chinese subjects. Eur. J. Drug Metab. Pharmacokinet. 45 (5), 601–609. 10.1007/s13318-020-00626-4 32474728 PMC7511473

[B13] Ferner M. Wachtlin D. Konrad T. Deuster O. Meinertz T. von Bardeleben S. (2016). Rationale and design of the RE-LATED AF--AFNET 7 trial: resolution of left atrial-appendage thrombus--effects of dabigatran in patients with atrial fibrillation. Clin. Res. Cardiol. 105 (1), 29–36. 10.1007/s00392-015-0883-7 26109251

[B14] Gombár C. Horvath G. Gálity H. Sisák K. Tóth K. (2014). Comparison of minor bleeding complications using dabigatran or enoxaparin after cemented total hip arthroplasty. Arch. Orthop. Trauma Surg. 134 (4), 449–457. 10.1007/s00402-014-1933-8 24488447

[B15] Holster I. L. Valkhoff V. E. Kuipers E. J. Tjwa E. T. (2013). New oral anticoagulants increase risk for gastrointestinal bleeding: a systematic review and meta-analysis. Gastroenterology 145 (1), 105–112.e15. 10.1053/j.gastro.2013.02.041 23470618

[B16] Izcovich A. Criniti J. M. Popoff F. Lu L. Wu J. Ageno W. (2020). Thrombolytics for venous thromboembolic events: a systematic review with meta-analysis. Blood Adv. 4 (7), 1539–1553. 10.1182/bloodadvances.2020001513 32289164 PMC7160254

[B17] Jafarzadeh-Esfehani R. Mostafa Parizadeh S. Sabeti Aghabozorgi A. Yavari N. Sadr-Nabavi A. Alireza Parizadeh S. (2020). Circulating and tissue microRNAs as a potential diagnostic biomarker in patients with thrombotic events. J. Cell. Physiol. 235 (10), 6393–6403. 10.1002/jcp.29639 32198752

[B18] Kim J. B. Joung H. J. Lee J. M. Woo J. S. Kim W. S. Kim K. S. (2016). Evaluation of the vascular protective effects of new oral anticoagulants in high-risk patients with atrial fibrillation (PREFER-AF): study protocol for a randomized controlled trial. Trials 17 (1), 422 10.1186/s13063-016-1541-8 27558002 PMC4997652

[B19] Laslett L. J. Alagona P. Jr. Clark B. A. 3rd Drozda J. P. Jr. Saldivar F. Wilson S. R. (2012). The worldwide environment of cardiovascular disease: prevalence, diagnosis, therapy, and policy issues: a report from the American College of Cardiology. J. Am. Coll. Cardiol. 60 (25 Suppl. l), S1–S49. 10.1016/j.jacc.2012.11.002 23257320

[B20] Liang H. Liang W. Zhao L. Chen D. Zhang J. Zhang Y. (2018). Robotic versus video-assisted lobectomy/segmentectomy for lung cancer: a meta-analysis. Ann. Surg. 268 (2), 254–259. 10.1097/SLA.0000000000002346 28628562

[B43] Lin L. Chu H. Murad M. H. Hong C. Qu Z. Cole S. R. (2018). Empirical comparison of publication bias tests in meta-analysis. J. Gen. Intern. Med. 33 (8), 1260–1267. 10.1007/s11606-018-4425-7 29663281 PMC6082203

[B21] Ma C. Riou França L. Lu S. Diener H. C. Dubner S. J. Halperin J. L. (2020). Stroke prevention in atrial fibrillation changes after dabigatran availability in China: the GLORIA-AF registry. J. Arrhythm 36 (3), 408–416. 10.1002/joa3.12321 32528565 PMC7279964

[B22] Mackman N. Bergmeier W. Stouffer G. A. Weitz J. I. (2020). Therapeutic strategies for thrombosis: new targets and approaches. Nat. Rev. Drug Discov. 19 (5), 333–352. 10.1038/s41573-020-0061-0 32132678

[B23] Majeed A. Hwang H. Eikelboom J. W. Connolly S. Wallentin L. Feuring M. (2016). Effectiveness and outcome of management strategies for dabigatran- or warfarin-related major bleeding events. Thromb. Res. 140, 81–88. 10.1016/j.thromres.2016.02.005 26908016

[B24] Mega J. L. Braunwald E. Wiviott S. D. Bassand J. Bhatt D. L. Bode C. (2012). Rivaroxaban in patients with a recent acute coronary syndrome. N. Engl. J. Med. 366 (1), 9–19. 10.1056/NEJMoa1112277 22077192

[B25] Oldgren J. Budaj A. Granger C. B. Khder Y. Roberts J. Siegbahn A. (2011). Dabigatran vs. placebo in patients with acute coronary syndromes on dual antiplatelet therapy: a randomized, double-blind, phase II trial. Eur. Heart J. 32 (22), 2781–2789. 10.1093/eurheartj/ehr113 21551462

[B26] Özler T. (2015). Comparison of switch therapy modalities (enoxaparine to rivaroxaban or dabigatran) with enoxaparine alone after hip and knee replacement; a non-blinded, randomised study. Acta Orthop. Traumatol. Turcica 49 (3), 255–259. 10.3944/AOTT.2015.14.0219 26200403

[B27] Peetermans M. Liesenborghs L. Peerlinck K. Wijngaerden E. Gheysens O. Goffin K. (2018). Targeting coagulase activity in staphylococcus aureus bacteraemia: a randomized controlled single-centre trial of staphylothrombin inhibition. Thromb. Haemostasis 118 (5), 818–829. 10.1055/s-0038-1639586 29614521

[B28] Pepe Ribeiro de Souza C. Bolzachini Santoni N. Gomes de Melo T. Jansen de Oliveira Figueiredo M. da Costa Darrieux F. C. Soares Piegas L. (2015). Cost-effectiveness and cost-utility analyses of dabigatran compared with warfarin in patients with nonvalvular atrial fibrillation and risk factors for stroke and systemic embolism within Brazilian private and public health care systems perspectives. Value Health Reg. Issues 8, 36–42. 10.1016/j.vhri.2015.02.003 29698169

[B29] Polzin A. Dannenberg L. Wolff G. Zeus T. Kelm M. Petzold T. (2018). Increased risk of myocardial infarction with dabigatran etexilate: fact or fiction? A critical meta-analysis from integrating randomized controlled trials and real-world studies: wine or spritzer?. Int. J. Cardiol. 270, 82 10.1016/j.ijcard.2018.07.020 30219540

[B30] Prandoni P. GhirarduzzI A. Prins M. H. Pengo V. Davidson B. L. Sørensen H. (2006). Venous thromboembolism and the risk of subsequent symptomatic atherosclerosis. J. Thromb. Haemostasis 4 (9), 1891–1896. 10.1111/j.1538-7836.2006.02058.x 16961597

[B31] Prandoni P. (2009). Venous and arterial thrombosis: two aspects of the same disease?. Clin. Epidemiol. 1, 1–6. 10.2147/clep.s4780 20865079 PMC2943163

[B32] Schleichert R. Goldner R. Dickfeld T. (2016). Palmoplantar pustular eruption due to dabigatran. Cutis 97 (5), E10–E11. 27274551

[B44] Schulman S. Kearon C. (2005). Subcommittee on control of anticoagulation of the scientific and standardization committee of the international society on thrombosis and haemostasis. Definition of major bleeding in clinical investigations of antihemostatic medicinal products in non-surgical patients. J. Thromb. Haemost. 3 (4), 692–4. 10.1111/j.1538-7836.2005.01204.x 15842354

[B33] Schulman S. Kearon C. Kakkar A. K. Mismetti P. Schellong S. Eriksson H. (2009). Dabigatran versus warfarin in the treatment of acute venous thromboembolism. N. Engl. J. Med. 361 (24), 2342–2352. 10.1056/NEJMoa0906598 19966341

[B34] Schulman S. Angerås U. Bergqvist D. Eriksson B. Lassen M. R. Fisher W. (2010). Definition of major bleeding in clinical investigations of antihemostatic medicinal products in surgical patients. J. Thromb. Haemostasis 8 (1), 202–204. 10.1111/j.1538-7836.2009.03678.x 19878532

[B35] Schulman S. Kakkar A. K. Goldhaber S. Z. Schellong S. Eriksson H. Mismetti P. (2014). Treatment of acute venous thromboembolism with dabigatran or warfarin and pooled analysis. Circulation 129 (7), 764–772. 10.1161/CIRCULATIONAHA.113.004450 24344086

[B36] Sipahi I. Celik S. Tozun N. (2014). A comparison of results of the US food and drug administration's mini-sentinel program with randomized clinical trials: the case of gastrointestinal tract bleeding with dabigatran. JAMA Intern Med. 174 (1), 150 10.1001/jamainternmed.2013.12217 24247291

[B37] Song Z. K. Cao H. Wu H. Wei Q. Tang M. Yang S. (2020). Current status of rivaroxaban in elderly patients with pulmonary embolism (review). Exp. Ther. Med. 19 (4), 2817–2825. 10.3892/etm.2020.8559 32256765 PMC7086161

[B38] Sterne J. A. Egger M. Smith G. D. (2001). Systematic reviews in health care: investigating and dealing with publication and other biases in meta-analysis. BMJ 323 (7304), 101–105. 10.1136/bmj.323.7304.101 11451790 PMC1120714

[B39] Wendelboe A. M. Raskob G. E. (2016). Global burden of thrombosis: epidemiologic aspects. Circ. Res. 118 (9), 1340–1347. 10.1161/CIRCRESAHA.115.306841 27126645

[B40] Wolberg A. S. Rosendaal F. R. Weitz J. I. Jaffer I. H. Agnelli G. Baglin T. (2015). Venous thrombosis. Nat. Rev. Dis. Primers 1, 15006 10.1038/nrdp.2015.6 27189130

[B41] Xue P. Ma Z. Liu S. (2019). Efficacy and safety of ginkgo leaf extract and dipyridamole injection for ischemic stroke: a systematic review and meta analysis. Front. Pharmacol. 10, 1403 10.3389/fphar.2019.01403 31866861 PMC6904941

[B42] Yasaka M. (2013). Direct oral thrombin inhibitor, "dabigatran". Nippon Rinsho 71 (1), 113–118. 23631181

